# Supplementary material for: CHIR99021 Maintenance of the Cell Stemness by Regulating Cellular Iron Metabolism
Source: Antioxidants (Basel). 2023 Feb 4;12(2):377. doi: 10.3390/antiox12020377 (PMC9952287; doi:10.3390/antiox12020377)
Supplement: Supplementary file 1 [file antioxidants-12-00377-s001.zip › antioxidants-2117105-supplementary.pdf]

Supplementary Figures

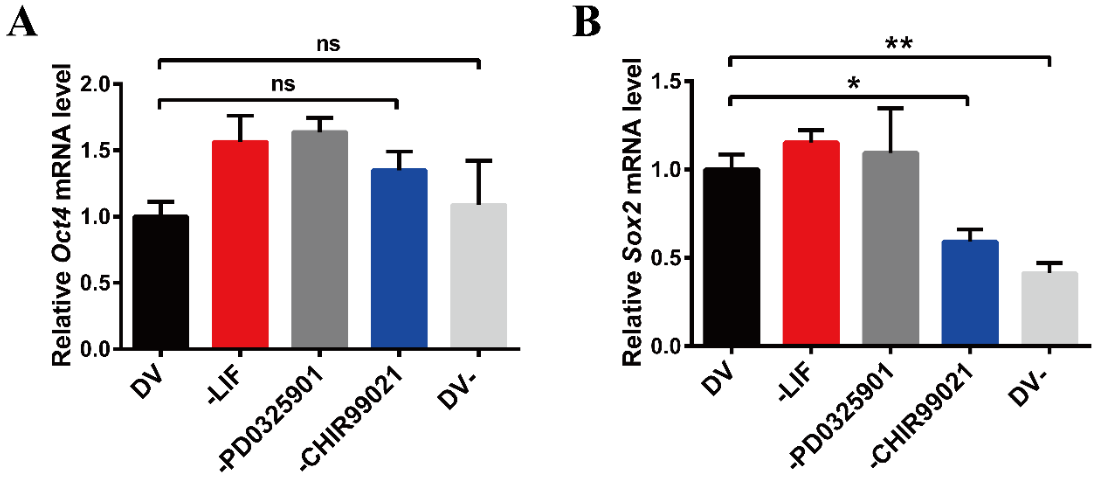

Figure S1. Effects of removal CHIR99021 on the expression of *Oct4* and *Sox2* genes. (A-B) qRT-PCR analysis of pluripotency genes *Oct4* (A) and *Sox2* (B) expression. DV, derivation medium; -(LIF, PD0325901 or CHIR99021), derivation medium lacking the indicated component; DV-, differentiation medium lacking LIF, PD0325901 and CHIR99021. The mRNA levels are normalized to GAPDH mRNA levels. The data are expressed as the mean ± SEM. *$p < 0.05$, **$p < 0.01$, ns, not significant.

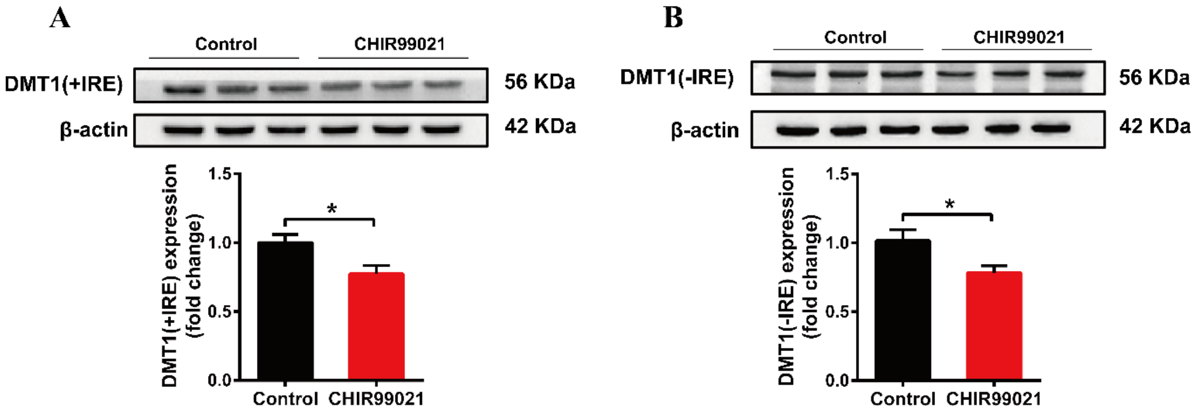

Figure S2. Expression of DMT1 in Neuro-2a cells treated with CHIR99021. (A-B) Western blot analysis and quantification of the expression of DMT1(+IRE) and DMT1(-IRE) protein. The relative expression levels are normalized to β-actin and then expressed as the fold of control group. The data are expressed as the mean ± SEM. *$p < 0.05$.
